# Simultaneous presentation of vulvar aggressive angiomyxoma and ipsilateral subcutaneous lipoma: a case report

**DOI:** 10.3389/fmed.2026.1967076

**Published:** 2026-10-14

**Authors:** Eiman Shalabna, Mariana Ordonez, Erika Gandelsman, Ariel Polonsky, Ilan Bruchim, Benjamin Feiner

**Affiliations:** 1 Ruth and Bruce Rappaport Faculty of Medicine, Technion—Israel Institute of Technology, Haifa, Israel; 2 Department of Obstetrics and Gynecology, Hillel Yaffe Medical Center, Hadera, Israel

**Keywords:** aggressive angiomyxoma, deep angiomyxoma, lipoma, soft tissue tumor, vulvar neoplasm

## Abstract

**Background:**

Aggressive angiomyxoma (AAM) is a rare, locally infiltrative mesenchymal neoplasm of the pelviperineal region that is frequently misdiagnosed owing to its slow growth and nonspecific presentation. Synchronous presentation with a second, unrelated large soft-tissue tumor has not previously been described.

**Case:**

We report a 53-year-old woman presenting with two sizeable, ipsilateral, left-sided masses: a pedunculated 13-cm vulvar lesion and a 30-cm multilobulated thigh mass. Ultrasound depicted the vulvar lesion as cystic; MRI favored AAM in the vulvar region and raised concern for liposarcoma in the thigh; initial biopsies were inconclusive. A multidisciplinary tumor board directed staged surgical excision. Histopathology confirmed vulvar AAM with estrogen receptor, progesterone receptor, and CD34 positivity and desmin and smooth muscle actin negativity, and a benign subcutaneous lipoma in the thigh, with no MDM2 amplification on fluorescence *in situ* hybridization. The postoperative course was uncomplicated.

**Conclusion:**

This unusual concurrent presentation illustrates the diagnostic challenges posed by AAM and underscores the value of advanced imaging, definitive histopathological confirmation, and coordinated multidisciplinary planning. Given the high risk of local recurrence, long-term surveillance remains essential.

## Introduction

Aggressive angiomyxoma (AAM) is a rare, slow-growing mesenchymal tumor that predominantly arises in the pelvic and perineal regions of women of reproductive age (1, 2). Because of its rarity, AAM is frequently mistaken for benign conditions such as Bartholin cysts, lipomas, or hernias (3). Although the term “aggressive” refers to its locally infiltrative growth pattern, distant metastasis is exceptionally rare. AAM nonetheless poses considerable diagnostic and therapeutic challenges, with local recurrence rates of 30%–70% reported even after wide local excision (4).

The synchronous occurrence of AAM with a second, unrelated soft-tissue tumor has not, to our knowledge, previously been reported. Here, we describe a patient with two large, ipsilateral, left-sided masses—a vulvar AAM and a subcutaneous thigh lipoma—that together produced a distinct diagnostic and therapeutic dilemma requiring multidisciplinary coordination.

## Case presentation

A 53-year-old woman, gravida 1 para 1, presented with a mass arising from the left vulva, first noticed three years earlier and progressively enlarging over the preceding six months. She additionally reported a swelling of the left thigh present for two years. She denied pain, fever, abnormal uterine bleeding, ulceration, dyspareunia, urinary symptoms, or difficulty with defecation. Her history was notable for longstanding anxiety and poor engagement with routine medical care, which had contributed to delayed evaluation.

On examination, a large, globular, soft, pedunculated mass arose from the left labia majora; it was non-tender and non-ulcerated. The left thigh showed a sizeable, multilobulated mass with similar soft-tissue characteristics (Figure 1).

**Figure 1:**
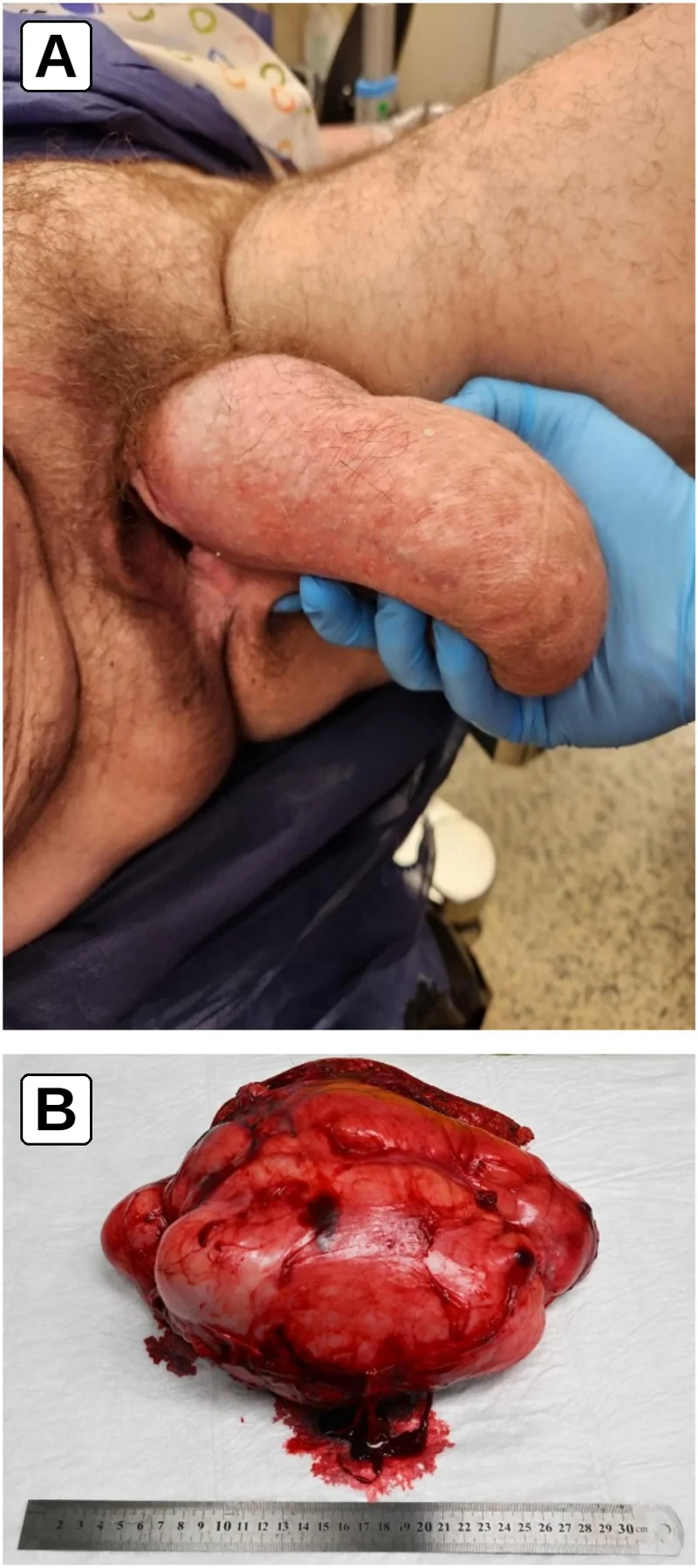
Clinical presentation. **(A)** Pedunculated 13-cm mass arising from the left labia majora. **(B)** Excised 30-cm multilobulated left thigh mass, shown with a ruler for scale.

Initial pelvic ultrasound showed a cystic-appearing vulvar lesion with viscous-appearing contents and no evidence of solid components, in addition to a normal endometrium (4.6 mm), a 20 × 17 mm anterior subserosal leiomyoma, and normal ovaries. Pelvic MRI demonstrated a 7 × 10 × 13 cm heterogeneous vulvar mass with gadolinium enhancement and a “swirled” T2 pattern, a finding strongly suggestive of AAM (Figure 2). MRI of the thigh showed a 16 × 11 × 30 cm multilobulated mass with thickened septations and focal necrosis, raising concern for liposarcoma (Figure 2). Fine-needle aspiration of both lesions was nondiagnostic; deep core biopsies obtained under general anesthesia suggested features consistent with AAM at both sites.

**Figure 2:**
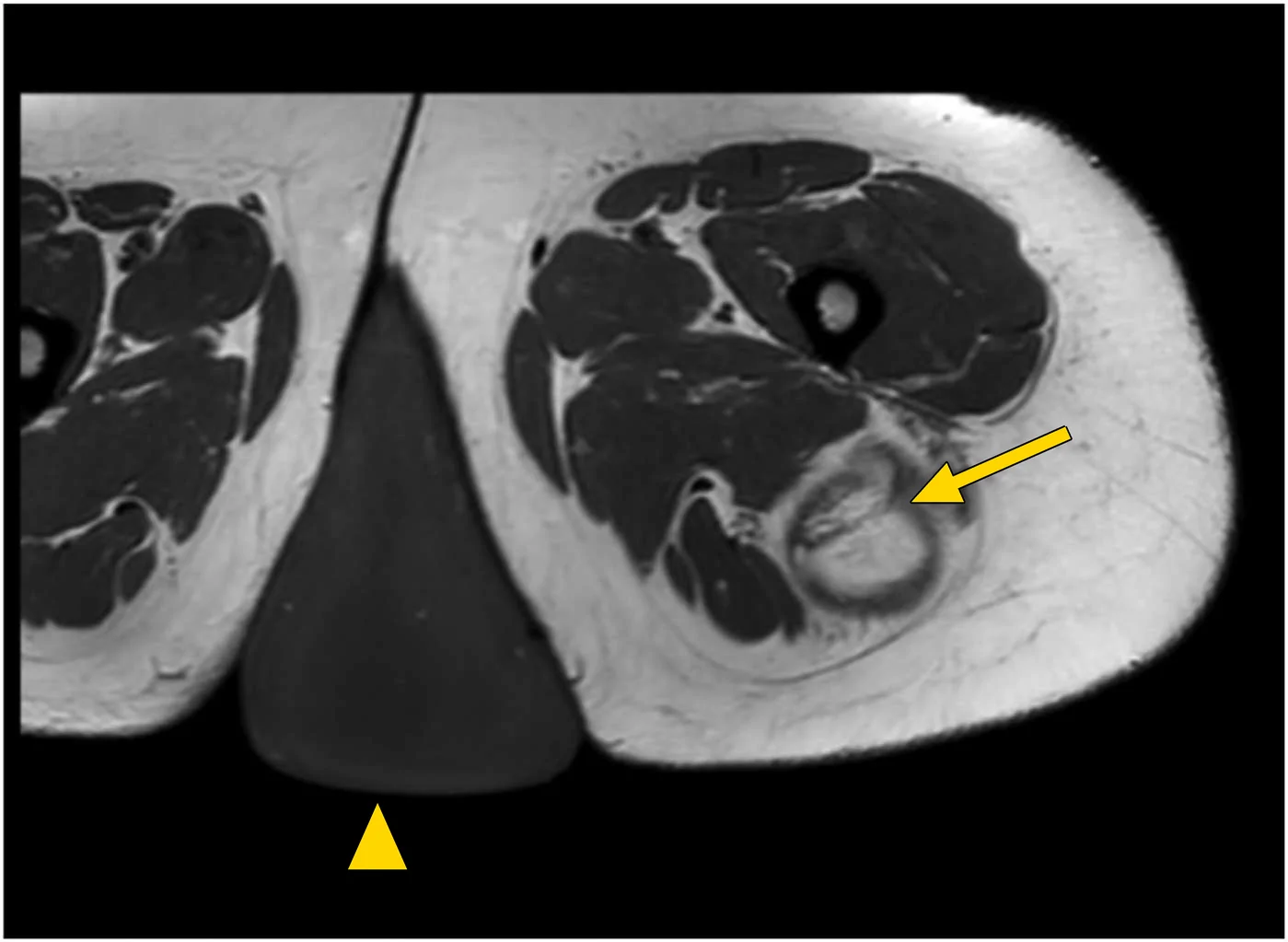
Axial MRI demonstrating both lesions. The left thigh mass is a well-defined, multilobulated soft-tissue mass with heterogeneous enhancement (arrow); the vulvar AAM appears as a heterogeneous lesion in the left vulvar region (arrowhead).

Management was directed by a multidisciplinary tumor board, which recommended staged excision. Because of its size and the radiological concern for malignancy, the thigh mass was excised first, by orthopedic oncology, with complete resection; final pathology confirmed a benign subcutaneous lipoma, composed of mature adipocytes without cytologic atypia or mitotic activity, with no MDM2 amplification on fluorescence *in situ* hybridization; the diagnosis was confirmed on expert pathology review. Two weeks later, the vulvar mass was excised via a vaginal approach, using a circular incision around the pedunculated base with careful dissection along the left paracervical space. A Penrose drain was placed and removed uneventfully on postoperative day two. Final pathology confirmed AAM: a non-circumscribed, paucicellular tumor composed of small oval, spindle and stellate cells within a loose myxoid stroma, with vessels of varying caliber and thin to thick, partly hyalinized walls, and without nuclear atypia or mitotic activity. Tumor cells were positive for estrogen receptor, progesterone receptor, and CD34, and negative for desmin and smooth muscle actin (Figure 3). HMGA2 immunohistochemistry and HMGA2 rearrangement studies were not performed.

**Figure 3:**
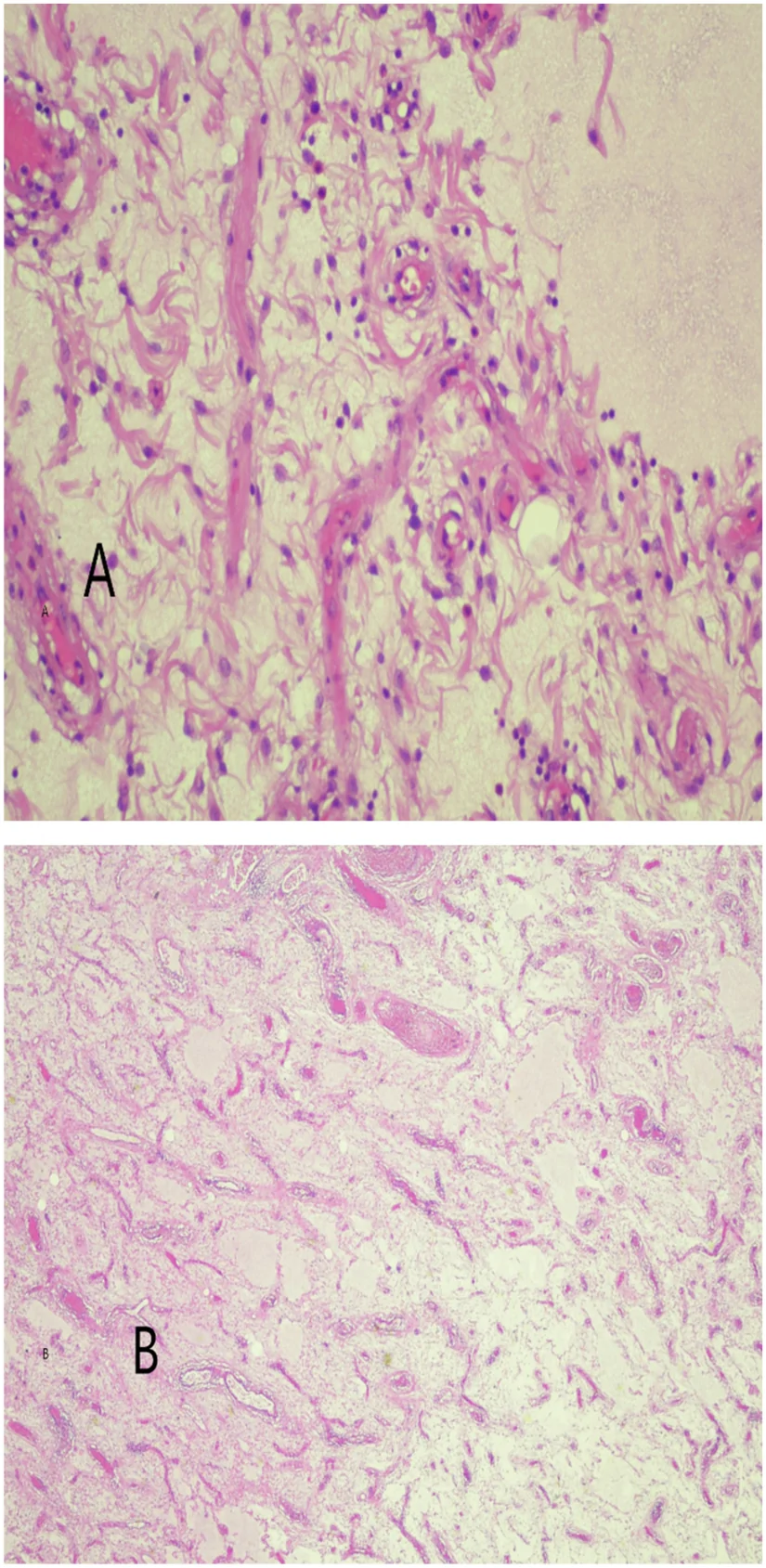
Hematoxylin and eosin staining of the vulvar mass. **(A)** Admixture of thin- and thick-walled vessels of varying caliber within a fibromyxoid stroma. **(B)** Spindle-shaped tumor cells within a myxoid stroma.

Despite repeated outreach, the patient—whose anxiety affected her adherence to follow-up—did not return for evaluation until nine months postoperatively. Examination at that visit showed well-healed incisions without evidence of recurrence. She declined hormonal therapy and was referred for ultrasound and MRI surveillance, with continued gynecologic oncology follow-up and support from the hospital social worker.

## Discussion

AAM is a rare, hormonally responsive mesenchymal tumor arising predominantly in the pelvic and perineal soft tissues of women, with local recurrence reported in 30%–70% of cases even after apparently complete excision (4). Distant metastasis is exceptionally uncommon, with only a small number of cases described in the literature (5, 6).

The synchronous presentation of two large, ipsilateral soft-tissue tumors—a vulvar AAM and a giant subcutaneous thigh lipoma—has not, to our knowledge, previously been reported. The coexistence of lesions differing in anatomical location, imaging characteristics, and suspected malignant potential required a coordinated diagnostic and surgical strategy: the thigh mass was prioritized because of its size and radiological concern for sarcoma, while the vulvar lesion was managed with a gynecologic approach tailored to the anticipated infiltrative behavior of AAM.

Preoperative diagnosis of AAM is difficult, as the tumor commonly mimics benign vulvar conditions such as Bartholin cysts or vulvar abscesses (2, 7, 8). MRI is the imaging modality of choice, with a “swirled” or “layered” appearance on T2-weighted sequences—reflecting the tumor's myxoid matrix—considered characteristic, though not specific (4, 9, 10). On ultrasound, AAM may appear as a hypoechoic or cystic-appearing mass because of its high myxoid water content, which can lead to misdiagnosis as a Bartholin cyst or other cystic vulvar lesion (10, 11). In our patient, ultrasound depicted the vulvar tumor as a cystic lesion without solid components and did not raise suspicion of a neoplasm, underscoring the limited specificity of ultrasound and the need for MRI when a vulvar mass is large or atypical. In this case, imaging correctly favored AAM at the vulvar site but raised concern for a distinct malignancy (liposarcoma) in the thigh, and fine-needle aspiration was nondiagnostic at both sites, underscoring the reliance on core biopsy and histopathology for definitive diagnosis.

AAM should also be distinguished from superficial angiomyxoma (cutaneous myxoma), a distinct entity despite the similar name. Superficial angiomyxoma arises in the dermis and subcutis of the head and neck, trunk, or genital region, affects both sexes, and may be associated with Carney complex (12). In the genital region it typically affects young women, with a mean age of approximately 21 years in one series (13). It is usually small, lobulated, and relatively well circumscribed, often contains scattered inflammatory cells, and is typically negative for hormone receptors; although it may recur locally, recurrences are non-destructive (12, 13). AAM, by contrast, occurs predominantly in women of reproductive age, with a peak incidence in the fourth decade, and is a deep-seated, often large, poorly circumscribed tumor of the pelviperineal soft tissues that expresses estrogen and progesterone receptors and carries a substantial risk of infiltrative local recurrence (1, 3, 4). Our patient's presentation at 53 years falls outside the typical age range for AAM; nevertheless, the large size, deep location, infiltrative growth, and hormone-receptor positivity of her tumor were characteristic of AAM rather than superficial angiomyxoma.

AAM is a hormonally responsive tumor that frequently expresses estrogen and progesterone receptors, consistent with its origin from mesenchymal cells with myofibroblastic differentiation (1–4, 9). Tumor growth may accelerate during pregnancy, a hormonal sensitivity that has prompted consideration of hormone-blocking therapies in unresectable or recurrent disease (4, 14, 15). Molecular studies have identified rearrangements involving HMGA2 at 12q13-15 in many cases, and immunohistochemical HMGA2 expression can aid diagnosis in equivocal cases (16). The ER, PR, and CD34 positivity observed here is consistent with AAM but not specific, as it is shared by other vulvovaginal stromal tumors. In this case, desmin negativity argued against angiomyofibroblastoma and myofibroblastoma, and the infiltrative, paucicellular architecture favored AAM over angiomyofibroblastoma and cellular angiofibroma (14). HMGA2 immunohistochemistry and rearrangement studies, which were not performed here, may help in equivocal cases (16), and MDM2 FISH was used to exclude atypical lipomatous tumor in the thigh lesion.

Surgical excision remains the cornerstone of AAM management, though no consensus exists on optimal margin strategy: some authors advocate wide excision with tumor-free margins to minimize recurrence, while others favor maximal safe resection to preserve function, particularly when radical surgery would compromise adjacent structures (1, 5, 11, 16). Given the tumor's hormone sensitivity, GnRH agonists have become a key adjunct, inducing hypoestrogenism and achieving an overall response rate exceeding 60% in a multicenter retrospective series (6). Aromatase inhibitors and selective estrogen receptor modulators, such as raloxifene and tamoxifen, are alternatives for patients who fail GnRH therapy (4). Radiotherapy and chemotherapy have limited efficacy given the tumor's low mitotic activity and are not routinely recommended; embolization has been used selectively to reduce vascularity before surgery (17).

Despite its infiltrative behavior, AAM carries an excellent overall survival prognosis; local recurrence remains the primary long-term concern, with rates that vary widely by series and length of follow-up and that can manifest many years after initial treatment (4, 5, 9). Long-term, often lifelong, surveillance is therefore recommended, typically with serial MRI at 6–12-month intervals initially, transitioning to annual imaging if no recurrence is detected (9–11). Preservation of reproductive and sexual function is an important consideration in treatment selection, particularly in younger women, and must be balanced against the side effects of prolonged hormonal therapy, including menopausal symptoms and bone loss (4).

This case reinforces that AAM should remain in the differential diagnosis of any slow-growing vulvar or pelvic mass, even when a second, unrelated soft-tissue tumor is present. Accurate diagnosis depends on correlating MRI findings with definitive histopathology, and management is best delivered by a multidisciplinary team experienced in rare pelvic and soft-tissue tumors. Given the substantial long-term recurrence risk, sustained surveillance and consistent patient engagement,particularly in patients with barriers to follow-up, remain essential to long-term outcomes.

## Conclusion

Synchronous presentation of vulvar AAM with an unrelated giant soft-tissue tumor is, to our knowledge, previously unreported, and illustrates the diagnostic complexity this tumor can present. Coordinated imaging, histopathological confirmation, and multidisciplinary surgical planning enabled complete excision of both lesions with an uncomplicated recovery. Given AAM's high recurrence risk, long-term surveillance and proactive follow-up support remain essential to patient care.

## Data Availability

The original contributions presented in the study are included in the article/Supplementary Material, further inquiries can be directed to the corresponding author.
